# Interactions between vascular burden and amyloid-β pathology on trajectories of tau accumulation

**DOI:** 10.1093/brain/awad317

**Published:** 2023-09-18

**Authors:** Emma M Coomans, Danielle van Westen, Alexa Pichet Binette, Olof Strandberg, Nicola Spotorno, Geidy E Serrano, Thomas G Beach, Sebastian Palmqvist, Erik Stomrud, Rik Ossenkoppele, Oskar Hansson

**Affiliations:** Radiology and Nuclear Medicine, Vrije Universiteit Amsterdam, Amsterdam UMC location VUmc, 1081HV Amsterdam, The Netherlands; Amsterdam Neuroscience, Brain Imaging, 1081HV Amsterdam, The Netherlands; Clinical Memory Research Unit, Department of Clinical Sciences Malmö, Lund University, SE-222 42 Lund, Sweden; Alzheimer Center Amsterdam, Neurology, Vrije Universiteit Amsterdam, Amsterdam UMC location VUmc, 1081HV Amsterdam, The Netherlands; Clinical Memory Research Unit, Department of Clinical Sciences Malmö, Lund University, SE-222 42 Lund, Sweden; Clinical Memory Research Unit, Department of Clinical Sciences Malmö, Lund University, SE-222 42 Lund, Sweden; Clinical Memory Research Unit, Department of Clinical Sciences Malmö, Lund University, SE-222 42 Lund, Sweden; Clinical Memory Research Unit, Department of Clinical Sciences Malmö, Lund University, SE-222 42 Lund, Sweden; Banner Sun Health Research Institute, Sun City, AZ 85351, USA; Banner Sun Health Research Institute, Sun City, AZ 85351, USA; Clinical Memory Research Unit, Department of Clinical Sciences Malmö, Lund University, SE-222 42 Lund, Sweden; Memory Clinic, Skåne University Hospital, SE-205 02 Malmö, Sweden; Clinical Memory Research Unit, Department of Clinical Sciences Malmö, Lund University, SE-222 42 Lund, Sweden; Memory Clinic, Skåne University Hospital, SE-205 02 Malmö, Sweden; Alzheimer Center Amsterdam, Neurology, Vrije Universiteit Amsterdam, Amsterdam UMC location VUmc, 1081HV Amsterdam, The Netherlands; Memory Clinic, Skåne University Hospital, SE-205 02 Malmö, Sweden; Amsterdam Neuroscience, Neurodegeneration, 1071HV Amsterdam, The Netherlands; Clinical Memory Research Unit, Department of Clinical Sciences Malmö, Lund University, SE-222 42 Lund, Sweden; Memory Clinic, Skåne University Hospital, SE-205 02 Malmö, Sweden

**Keywords:** vascular burden, vascular risk, amyloid-β, tau, Alzheimer’s disease

## Abstract

Cerebrovascular pathology often co-exists with Alzheimer’s disease pathology and can contribute to Alzheimer’s disease-related clinical progression. However, the degree to which vascular burden contributes to Alzheimer’s disease pathological progression is still unclear. This study aimed to investigate interactions between vascular burden and amyloid-β pathology on both baseline tau tangle load and longitudinal tau accumulation.

We included 1229 participants from the Swedish BioFINDER-2 Study, including cognitively unimpaired and impaired participants with and without biomarker-confirmed amyloid-β pathology. All underwent baseline tau-PET (^18^F-RO948), and a subset (*n* = 677) underwent longitudinal tau-PET after 2.5 ± 1.0 years. Tau-PET uptake was computed for a temporal meta-region-of-interest. We focused on four main vascular imaging features and risk factors: microbleeds; white matter lesion volume; stroke-related events (infarcts, lacunes and haemorrhages); and the Framingham Heart Study Cardiovascular Disease risk score. To validate our *in vivo* results, we examined 1610 autopsy cases from an Arizona-based neuropathology cohort on three main vascular pathological features: cerebral amyloid angiopathy; white matter rarefaction; and infarcts. For the *in vivo* cohort, primary analyses included age-, sex- and *APOE* ɛ4-corrected linear mixed models between tau-PET (outcome) and interactions between time, amyloid-β and each vascular feature (predictors). For the neuropathology cohort, age-, sex- and *APOE* ɛ4-corrected linear models between tau tangle density (outcome) and an interaction between plaque density and each vascular feature (predictors) were performed.

In cognitively unimpaired individuals, we observed a significant interaction between microbleeds and amyloid-β pathology on greater baseline tau load (β = 0.68, *P* < 0.001) and longitudinal tau accumulation (β = 0.11, *P* < 0.001). For white matter lesion volume, we did not observe a significant independent interaction effect with amyloid-β on tau after accounting for microbleeds. In cognitively unimpaired individuals, we further found that stroke-related events showed a significant negative interaction with amyloid-β on longitudinal tau (β = −0.08, *P* < 0.001). In cognitively impaired individuals, there were no significant interaction effects between cerebrovascular and amyloid-β pathology at all. In the neuropathology dataset, the *in vivo* observed interaction effects between cerebral amyloid angiopathy and plaque density (β = 0.38, *P* < 0.001) and between infarcts and plaque density (β = −0.11, *P* = 0.005) on tau tangle density were replicated.

To conclude, we demonstrated that cerebrovascular pathology—in the presence of amyloid-β pathology—modifies tau accumulation in early stages of Alzheimer’s disease. More specifically, the co-occurrence of microbleeds and amyloid-β pathology was associated with greater accumulation of tau aggregates during early disease stages. This opens the possibility that interventions targeting microbleeds may attenuate the rate of tau accumulation in Alzheimer’s disease.

## Teaser

Coomans *et al*. investigate the effect of interactions between cerebrovascular burden and amyloid-β load on trajectories of tau accumulation, and find that the co-occurrence of cerebral microbleeds and amyloid-β pathology is associated with greater accumulation of tau aggregates during early disease stages.

## Introduction

Neuropathological studies have demonstrated that Alzheimer’s disease (AD) pathology [i.e. amyloid-β (Aβ) plaques and tau neurofibrillary tangles] frequently co-exists with cerebrovascular pathology.^1-6^ The prevalence of both cerebrovascular and AD pathology increases with older age, and cerebrovascular pathology has been shown to contribute to the AD clinical syndrome.^7-11^ For example, studies have demonstrated that cerebrovascular pathology may lower the threshold for dementia at a given burden of AD pathology^1,8-10^ and that cerebrovascular and Aβ pathology may have an additive or interactive effect on cognitive decline.^12-15^ Although vascular burden thus seems to contribute to AD-related clinical progression, it remains less clear to what extent vascular burden can contribute to AD pathological progression.

Previous studies showed that increased vascular burden (e.g. cerebrovascular pathology, vascular disease or vascular risk) was associated with increased hyper-phosphorylated tau, either independently or in interaction with Aβ.^16-22^ However, other studies observed no significant associations between vascular burden and tau pathology.^23-26^ Part of the heterogeneity in current literature may be related to differences in the operationalization of vascular burden, which ranged from neuropathological findings to *in vivo* MRI measures of cerebrovascular pathology to measures of (general) vascular risk and health. Moreover, it is important to take into account when vascular burden was measured (e.g. mid-life versus late-life). Furthermore, most previous studies focused solely on individuals without dementia, and some cohorts with stringent exclusion criteria related to the presence of cardiovascular or cerebrovascular disease may have a better vascular health compared to the general population. It is of importance to get a better understanding of whether vascular burden acts as a modifier of tau accumulation in both cognitively unimpaired and impaired individuals, since tau pathology is closely associated to cognitive decline and clinical progression across the entire AD continuum.^27-29^ This could improve our understanding of the pathophysiological mechanisms underlying AD dementia and thereby aid in the development of therapeutic strategies.

Therefore, the primary aim of the current study was to investigate whether cerebrovascular pathological measures on MRI [microbleeds, white matter lesion (WML) volume, lacunes, infarcts and haemorrhages] and vascular risk [the Framingham Heart Study Cardiovascular Disease (FHS-CVD) risk score] interact with Aβ on both baseline tau tangle load and longitudinal tau accumulation. To this end, we included 1229 participants with and without cognitive impairment from the Swedish BioFINDER-2 Study. In an effort to validate our primary results observed *in vivo*, we additionally included 1610 autopsy cases from the Arizona Study of Aging and Neurodegenerative Disorders cohort with available neuropathological data on cerebrovascular pathology [white matter (WM) rarefaction, cerebral amyloid angiopathy (CAA) and infarcts].^30^

## Materials and methods

### Participants

We included participants from the longitudinal Swedish BioFINDER-2 Study (NCT03174938). Participants eligible for inclusion in the present study were: (i) Aβ-negative cognitively unimpaired (CU) participants; (ii) Aβ-positive CU participants; (iii) Aβ-negative participants with mild cognitive impairment (MCI) or Aβ-negative non-AD dementia [grouped into one Aβ-negative cognitively impaired (CI) group]; and (iv) Aβ-positive participants with MCI or Aβ-positive AD dementia (grouped into one Aβ-positive CI group). Furthermore, participants had to be ≥50 years of age, have Aβ-status available (described below), have undergone at least one baseline tau-PET scan, and have data available on at least one vascular factor (described below) and on all major covariates of interest (age, sex and *APOE* ɛ4 carriership). Aβ-status (negative/positive) was defined using a previously established cut-off for neocortical global Aβ-PET (^18^F-flutemetamol) standardized uptake value ratio (SUVr) for all CU and MCI participants,^31^ and using previously established cut-offs for the CSF Aβ_42_/Aβ_40_ ratio (Roche NeuroToolKit)^32^ for all participants with dementia (since Aβ-PET is not part of the standard BioFINDER-2 research protocol for participants with dementia). We excluded one CU participant with a brain tumour and 14 autosomal dominant mutation-carrying participants (*C9orf72*, *GRN* and *MAPT* mutation carriers). We also excluded one Aβ-negative CU participant with very high tau-PET binding (Supplementary Fig. 1) which was identified as an outlier in statistical analyses within the Aβ-negative CU group. Upon further inspection, this participant was discordant on Aβ-PET (negative) and CSF Aβ_42_/Aβ_40_ (positive). Since we were not able to clearly define this participant as Aβ-negative and since this participant acted as a statistical outlier, we chose to exclude this participant from the analyses. The resulting *n* = 1229 participants included in the current study were enrolled in BioFINDER-2 between May 2017 and September 2022.

CU participants did not have significant neurological or psychiatric illnesses, and did not fulfil criteria for MCI or any dementia according to Diagnostic and Statistical Manual of Mental Disorders, Fifth Edition (DSM-5; mild or major neurocognitive disorder) clinical criteria.^33^ More detailed inclusion criteria have been described previously.^31^ Aβ-positive MCI and AD dementia participants fulfilled DSM-5 clinical criteria for, mild cognitive disorder or major cognitive impairment due to AD,^33^ respectively, and were Aβ-positive in accordance with the updated National Institute on Aging and Alzheimer’s Association (NIA-AA) criteria.^34^ The Aβ-negative CI group consisted of MCI or dementia participants with a variety of aetiologies as listed in Supplementary Table 1. Exclusion criteria for all diagnostic groups were having significant unstable systemic illness or organ failure that made it difficult to participate in the study, significant current alcohol or substance misuse, and refusing lumbar puncture or neuroimaging.

Written informed consent was obtained from all participants prior to entering the study. Ethical approval was obtained from the Regional Ethical Committee in Lund, Sweden. Approval for PET imaging was obtained from the Swedish Medicines and Products Agency and the local Radiation Safety Committee at Skåne University Hospital in Sweden.

### MRI measures of cerebrovascular pathology

MRI was performed on a 3 T Siemens MAGNETOM Prisma scanner equipped with a 64-channel receiver coil array (Siemens Healthcare). Sequences included a whole-brain T1-weighted anatomical magnetization-prepared rapid gradient echo sequence [MPRAGE; repetition time (TR) = 1900 ms, echo time (TE) = 2.54 ms, voxel size = 1 × 1 × 1 mm^3^], a T2-weighted fluid-attenuated inversion recovery sequence (FLAIR; TR = 5000 ms, TE = 393 ms, same resolution as for the T1-weighted image) and a 3D, multi gradient-echo pulse sequence (TR = 24 ms, TEs = 5.00, 8.80, 12.60, 16.40 and 20.20 ms with monopolar/fly-back readout gradients, flip angle = 15°, voxel size = 0.8 × 0.8 × 1.4 mm^3^). White matter lesions were segmented by the lesion growth algorithm as implemented in the LST toolbox version 3.0.0 (www.statistical-modelling.de/lst.html) for statistical parametric mapping.^35^ Global WML volume was quantified, adjusted for intracranial volume (ICV) by taking the ratio (resulting in a global WML volume percentage of ICV) and log-transformed to improve normality. Microbleeds, lacunes, infarcts and haemorrhages were defined by visual read by a trained and experienced neuroradiologist (D.W.). The 3D multi gradient-echo pulse sequence images with the strongest susceptibility weighting (TE 20.20 ms) were used to assess the number and location of microbleeds in line with established criteria.^36^ For analyses, we dichotomized the number of microbleeds into 0 (absent) and ≥1 (present). The location of microbleeds was dichotomized into lobar (at least one microbleed in a lobar region) and non-lobar (microbleeds restricted to non-lobar regions). The number of lacunes, infarcts and haemorrhages were assessed on T2 FLAIR sequences.^37^ Owing to the relatively low prevalence of each of these cerebrovascular events, we grouped lacunes, infarcts and haemorrhages into one ‘stroke-related events’ group. For analyses, we dichotomized stroke-related events into 0 (absence of lacunes, infarcts and haemorrhages) and ≥1 (presence of lacunes, infarcts and/or haemorrhages). Information on WML volume was missing for *n* = 13 participants and microbleeds was missing for *n* = 53 participants.

### Vascular risk score

We used the office-based FHS-CVD risk score as a measure of vascular risk.^38^ The office-based FHS-CVD risk score is a sex-specific multi-variable weighted risk score based on age, body mass index (BMI), systolic blood pressure [SBP, adjusted for the use of antihypertensive treatment (dichotomous)], diabetes status (dichotomous) and current cigarette smoking (dichotomous). The FHS-CVD risk score (expressed as a percentage) provides a probability estimate for cardiovascular events (including ischaemic stroke, haemorrhagic stroke, transient ischaemic attack, coronary death, myocardial infarction, coronary insufficiency, angina, peripheral artery disease and heart failure) occurring within 10 years of risk assessment. The FHS-CVD risk score was missing for *n* = 327 participants.

### PET measures of amyloid-β and tau pathology

All participants underwent baseline tau-PET (^18^F-RO948) acquired 70 to 90 min post-injection. A subset of *n* = 677 participants [677/1229 (55.1%)] had 2.5 ± 1.0 year longitudinal ^18^F-RO948 PET available. A total of 2153 scans were included (range: 1–5 scans per participant, mean: 1.75 scans per participant). Baseline Aβ-PET (^18^F-flutemetamol), acquired 90 to 110 min post-injection, was available for all CU (*n* = 542), all MCI (*n* = 333) and a small subset of dementia (*n* = 16) participants (since Aβ-PET is not part of the standard BioFINDER-2 research protocol for participants with dementia).

^18^F-RO948 and ^18^F-flutemetamol PET images were acquired on digital GE Discovery MI PET/CT scanners and preceded by a low-dose CT scan for attenuation correction purposes. PET images were processed according to standardized pipelines as described previously.^39^ The T1-weighted MPRAGE was used for PET image co-registration and template normalization. SUVr images were created using the inferior cerebellum as the reference region for ^18^F-RO948, and using the pons as the reference region for ^18^F-flutemetamol. Our primary outcome measure was ^18^F-RO948 SUVr in the commonly used temporal meta-region-of-interest (meta-ROI), consisting of a volume-weighted average of the bilateral entorhinal cortex, amygdala, parahippocampal gyrus, fusiform gyrus, and the middle and inferior temporal gyrus.^40^ For sensitivity analyses, we additionally examined ^18^F-RO948 SUVr in a late-stage tau-region corresponding to Braak stages V–VI (including parietal, frontal, occipital and superior temporal regions^41^) in addition to the temporal meta-ROI that reflects early-to-intermediate stage tau pathology. For statistical models including ^18^F-flutemetamol PET, we used neocortical ^18^F-flutemetamol SUVr, which consisted of a commonly used volume-weighted average of the bilateral prefrontal, lateral temporal, parietal, anterior cingulate and posterior cingulate/precuneus.^31^

### Neuropathological validation cohort

To validate our *in vivo* observed findings, we included neuropathological data from 1610 participants who donated their brain to the Arizona Study of Aging and Neurodegenerative Disorders/Brain and Body Donation Program study.^22,30^ For visualization purposes, we labelled participants as low-Aβ or high-Aβ based on the Consortium to Establish a Registry for Alzheimer’s Disease (CERAD) score for Aβ plaques.^42^ Participants with none-to-sparse Aβ plaques were labelled as low-Aβ, and participants with moderate-to-frequent Aβ plaques were labelled as high-Aβ. For statistical analyses, we used a semi-quantitative score for total Aβ plaque density and total tau neurofibrillary tangle density. Both Aβ plaque density and tau neurofibrillary tangle density were graded and staged at standard sites in frontal, temporal and parietal lobes, hippocampal CA1 and (trans)entorhinal regions using a semi-quantitative score of 0–3 based on the CERAD templates. This yielded a maximum total score of 15 for both plaques and tangles. We included three main neuropathological measures of cerebrovascular pathology corresponding to measures included in the Swedish BioFINDER-2 Study: (i) a dichotomized measure of WM rarefaction (defined as present if there was mentioning of WM rarefaction); (ii) a dichotomized measure of CAA; and (iii) a dichotomized measure of infarcts (defined as present if there was mentioning of any sort of infarct). In a subset of 1109 participants, we also had a semi-quantitative score of CAA available, which was rated similarly as the semi-quantitative score for total Aβ plaque and tau tangle density (maximum score of 15). We excluded cases with missing data on key variables for statistical analyses (age, sex, *APOE* ɛ4 status, plaque density, tangle density, WM rarefaction, CAA and infarcts).

### Statistical analyses

For primary analyses in participants from the Swedish BioFINDER-2 Study, three measures of cerebrovascular pathology [i.e. microbleeds (dichotomous), WML volume (continuous, adjusted for ICV and log-transformed) and stroke-related events (dichotomous)] and one measure of vascular risk [i.e. the FHS-CVD risk score (continuous)] were used. Secondary analyses included microbleed location (lobar versus non-lobar, with 0 microbleeds as reference group) and subcomponents of the FHS-CVD risk score [age, sex, BMI, SBP (adjusted for use of anti-hypertensive treatment), diabetes status and current smoking status].

Demographical characteristics between Aβ-positive and Aβ-negative participants at the same cognitive stage were compared using *t*-tests for continuous variables and χ^2^ tests for categorical variables. We tested associations of age and sex (predictors, separate models) with each vascular factor (outcomes, separate models) using linear or logistic regressions. We also established whether vascular risk (predictor) was associated with cerebrovascular pathology (outcomes, separate models) using linear or logistic regressions.

To investigate whether cerebrovascular pathology was observed more often in Aβ-positive and/or cognitively impaired participants compared to Aβ-negative and/or cognitively unimpaired participants, linear or logistic regressions between cerebrovascular pathology (outcomes, separate models) and cognitive stage by Aβ-status (predictor) were performed, adjusted for age, sex and *APOE* ɛ4 carriership. We used estimated marginal means for between-group *post hoc* pairwise comparisons, and adjusted *P*-values for multiple comparisons using the false discovery rate (FDR) correction method following the Benjamini–Hochberg procedure (adjusted for six comparisons).

To investigate the interaction effect between baseline vascular burden and baseline Aβ pathology (predictors, separate models per vascular factor) on trajectories of tau accumulation (outcome), we used linear mixed effects models with random intercepts (1 | participant). Adding a random slope (time | participant) to the models did not improve model fit based on Akaike information criterion and the χ^2^ statistic (*P* > 0.05). Models were adjusted for age, sex and *APOE* ɛ4 carriership, as well as for their interaction with time. Time reflected time (in years) from baseline tau-PET. Continuous variables were scaled (*z*-scored) within each model. All participants, including those with only baseline tau-PET data, were included in the models. Models were performed in the total sample (in which we additionally corrected for cognitive stage and its interaction with time) as well as separately in CU and CI participants. Because Aβ-PET was missing for ±95% of participants with dementia, models for the total sample and models restricted to CI participants included dichotomous Aβ-status (positive/negative, based on PET for CU and MCI participants, and based on CSF for dementia participants), whereas models restricted to CU participants included a continuous measure of neocortical Aβ-PET SUVr, since this is a more accurate reflection of Aβ load. To ensure that results in the CI group were unaffected by the use of a dichotomous measure for Aβ-pathology, we performed sensitivity analyses using neocortical Aβ-PET SUVr in the CI subset that had Aβ-PET available. We had two main effects of interest: the fixed effect of Vascular factor × Aβ (which was interpreted as the interaction effect on baseline tau); and the fixed effect of Vascular factor × Aβ × Time (which was interpreted as the interaction effect on longitudinal tau). *P*-values were adjusted for multiple comparisons using the FDR correction method, which was applied separately for baseline effects and longitudinal effects within each group and adjusted for the number of primary analyses. We performed additional linear mixed effects models in each group independently (Aβ-negative CU, Aβ-positive CU, Aβ-negative CI and Aβ-positive CI; thereby removing the interaction term with Aβ) to help interpret the observed interaction effects.

To validate our findings in the Arizona-based neuropathology cohort, we performed linear regressions between tau tangle density (outcome) and an interaction between plaque density and each measurement of cerebrovascular pathology (CAA, WM rarefaction and infarcts, separate models per vascular factor), corrected for age at death, sex and *APOE* ɛ4 carriership.

All analyses were performed in R version 4.2.1. A *P*-value <0.05 was considered statistically significant.

## Results

### Participants

Participant characteristics are shown in Table 1. A total of 1229 participants were included from the Swedish BioFINDER-2 Study, among which 397 Aβ-negative CU, 145 Aβ-positive CU, 257 Aβ-negative CI and 430 Aβ-positive CI participants. Compared to Aβ-negative CU participants, Aβ-positive CU participants were older (*P* < 0.001), more often *APOE* ɛ4 carriers (*P* < 0.001) and had lower Mini-Mental State Examination (MMSE) scores (*P* = 0.001). Compared to Aβ-negative CI participants, Aβ-positive CI participants were older (*P* = 0.03), more often female (*P* < 0.001), more often *APOE* ɛ4 carriers (*P* < 0.001), had higher education (*P* = 0.01) and had lower MMSE scores (*P* < 0.001). Tau-PET SUVr in the temporal meta-ROI was significantly higher in Aβ-positive CU and CI participants compared to Aβ-negative participants at the same cognitive stage (both *P* < 0.001).

**Table 1:** Demographics BioFINDER-2 cohort

	Cognitively unimpaired (CU)		Cognitively impaired (CI)	
	CU Aβ-negative	CU Aβ-positive	CI Aβ-negative (MCI/non-AD dementia)	CI Aβ-positive (MCI/AD dementia)
*n*	397	145	257	430
Age, years	66.1 ± 10.0	71.4 ± 9.0^a^	71.6 ± 8.3	72.9 ± 7.1^b^
Sex, *n* female (%)	212 (53.4)	79 (54.5)	90 (35.0)	223 (51.9)^b^
*APOE* ɛ4 status, *n* carrier (%)	155 (39.0)	102 (70.3)^a^	59 (23.0)	306 (71.2)^b^
Education, years	12.8 ± 3.4	12.6 ± 3.8	11.8 ± 3.7	12.6 ± 4.3^b^
MMSE	29.0 ± 1.2	28.6 ± 1.5^a^	26.0 ± 3.2	23.7 ± 4.5^b^
**Cerebrovascular pathology**				
WML volume (% of ICV)	0.19 ± 0.37	0.25 ± 0.33	0.45 ± 0.74	0.44 ± 0.54
≥1 Microbleeds, *n* present (%)	27 (7.0)	23 (16.1)^a^	40 (16.9)	107 (26.0)^b^
Microbleed location, *n* present lobar (%)	17 (63.0)	20 (87.0)^a^	26 (65.0)	86 (80.4)^b^
≥1 Stroke-related events, *n* present (%)	43 (10.8)	18 (12.4)	58 (22.6)	80 (18.6)
**Vascular risk**				
FHS-CVD risk score	27.8 ± 19.0	32.5 ± 18.9^a^	36.8 ± 20.6	35.3 ± 18.5
Body mass index	27.0 ± 4.3	25.6 ± 3.5^a^	26.5 ± 4.5	25.1 ± 3.8^b^
SBP	143.0 ± 18.5	146.1 ± 18.0	145.1 ± 21.0	147.7 ± 19.0
Antihypertension drugs, *n* yes (%)	138 (40.2)	55 (45.8)	93 (45.4)	158 (48.2)
Diabetes, *n* yes (%)	36 (10.5)	13 (10.8)	38 (18.5)	43 (13.1)
Current smoker, *n* yes (%)	21 (5.3)	9 (6.4)	19 (7.8)	16 (3.9)
**Aβ-PET and tau-PET**				
Global Aβ-PET SUVr	0.47 ± 0.02	0.72 ± 0.13^a^	0.47 (0.03)	0.79 (0.14)^b^
Temporal meta-ROI tau-PET SUVr	1.14 ± 0.09	1.30 ± 0.29^a^	1.16 ± 0.11	1.84 ± 0.65^b^
*n* with longitudinal tau-PET, *n* (%)	276 (69.5)	93 (64.1)	121 (47.1)	187 (43.5)
Tau-PET follow-up time, years	2.6 ± 1.0	2.6 ± 1.0	2.2 ± 0.8	2.0 ± 0.7^b^

Shown is mean ± standard deviation, unless specified otherwise. Aβ-status (negative/positive) was based on Aβ-PET for cognitively unimpaired (CU) and mild cognitive impairment (MCI) participants and based on CSF Aβ_42_/Aβ_40_ ratio for dementia participants. Education was missing for *n* = 29; Mini-Mental State Examination (MMSE) for *n* = 10; white matter lesion (WML) volume for *n* = 13; microbleeds for *n* = 53; FHS-CVD risk score for *n* = 327; body mass index for *n* = 118; systolic blood pressure (SBP) for *n* = 73; antihypertension drugs for *n* = 233; diabetes for *n* = 233; current smoker for *n* = 44; and Aβ-PET for *n* = 338 [Aβ-PET was missing only in the cognitively impaired (CI) group]. AD = Alzheimer’s disease; FHS-CVD = Framingham Heart Study Cardiovascular Disease; ICV = intracranial volume; ROI = region of interest; SUVr = standardized uptake value ratio.

^a^CU Aβ+ significantly different from CU Aβ−.

^b^CI Aβ+ significantly different from CI Aβ−.

### Prevalence of cerebrovascular pathology

In the total cohort, microbleeds were observed in 16.8% of participants (197/1176) and stroke-related events were observed in 16.2% of participants (199/1229). These participants were mostly non-overlapping, with 6.7% of participants (79/1176) showing both microbleeds and stroke-related events. WML volume (expressed as a percentage of intracranial volume) ranged from 0% to 6.1% (mean: 0.34%). WML volume, microbleeds and stroke-related events were all significantly associated with older age (all *P* < 0.001) (Supplementary Fig. 2). Microbleeds and stroke-related events were associated with male sex (both *P* < 0.01), but WML volume was not (*P* = 0.48) (Supplementary Fig. 2).

**Figure 1:**
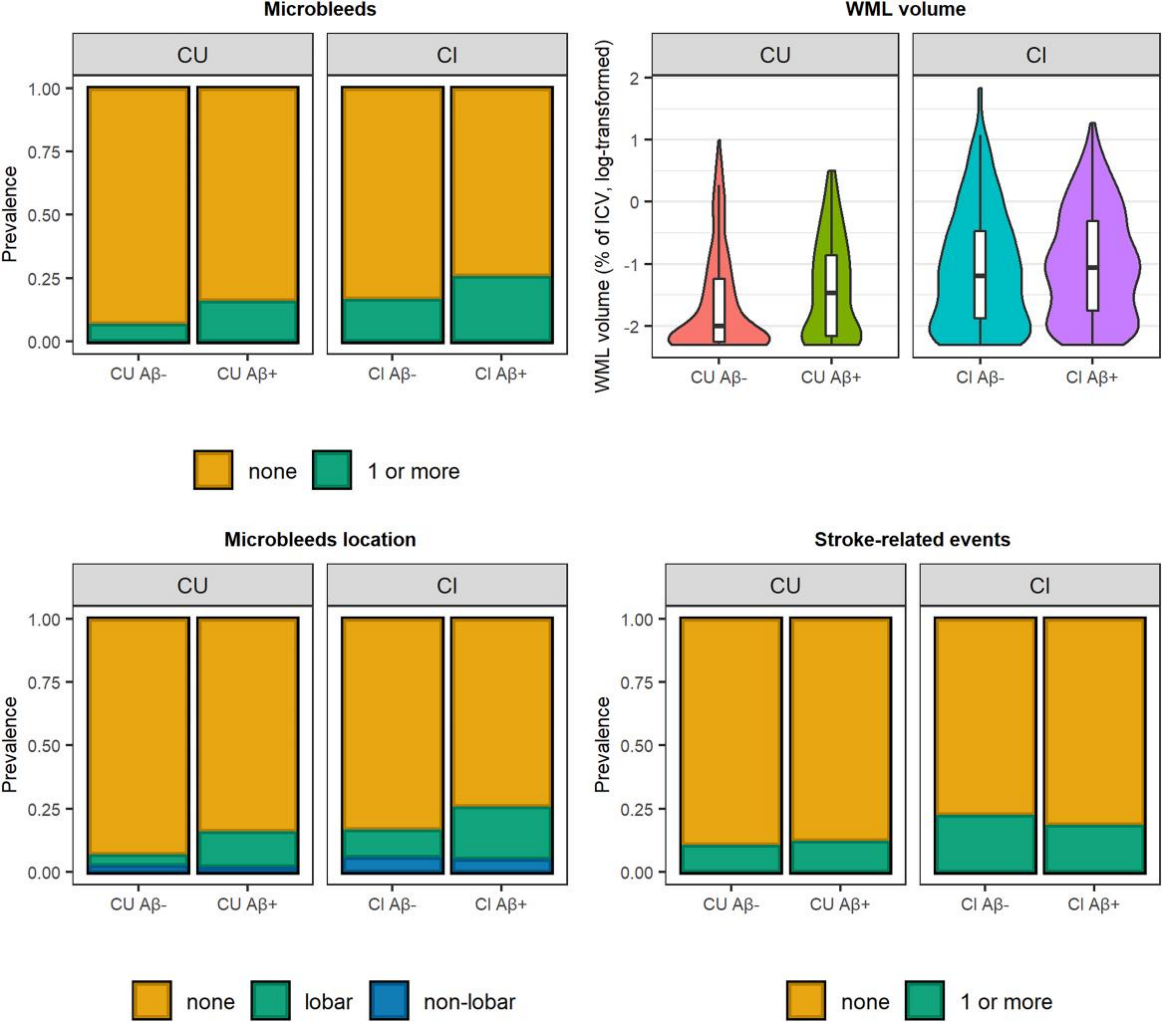
**Cerebrovascular pathology per group in the Swedish BioFINDER-2 study.** Shown are white matter lesion (WML) volume (adjusted for intracranial volume and log-transformed), the prevalence of having one or more microbleeds (as well as their location), and the prevalence of having one or more stroke-related events for each group. Estimates and *P*-values for group comparisons are shown in Supplementary Table 2. Amyloid-β (Aβ)-positive participants showed significantly more (lobar) microbleeds compared to Aβ-negative participants at the same cognitive stage. Also, both Aβ-positive and Aβ-negative cognitively impaired (CI) participants showed significantly more (lobar) microbleeds compared to Aβ-negative cognitively unimpaired (CU) participants. Furthermore, CI participants showed significantly larger WML volume compared to CU participants, irrespective of Aβ-status.

We first tested whether cerebrovascular pathology was observed more often in Aβ-positive compared to Aβ-negative participants, and in impaired compared to unimpaired participants (Fig. 1). Corrected for age, sex and *APOE* ɛ4 carriership, Aβ-positive participants showed more microbleeds compared to Aβ-negative participants at the same cognitive stage (CU: β = 0.66, *P* = 0.04; CI: β = 0.51, *P* = 0.04). These differences became even more apparent when restricting the analyses to lobar microbleeds (CU: β = 1.03, *P* = 0.01; CI: β = 0.74, *P* = 0.01). There were no significant differences between Aβ-positive and Aβ-negative participants at the same cognitive stage in WML volume and stroke-related events (all *P* > 0.05; Supplementary Table 2). When comparing CI to CU participants, we observed that both Aβ-positive and Aβ-negative CI participants showed a significantly higher prevalence of (lobar) microbleeds compared to Aβ-negative CU participants (Supplementary Table 2). Furthermore, CI participants showed significantly larger WML volumes compared to CU participants, irrespective of participants’ Aβ-status (estimate range: 0.24–0.36, all *P* < 0.01) (Supplementary Table 2). No significant differences were observed in stroke-related events.

### Role of cerebrovascular pathology in the association between amyloid-β and tau

**Figure 2:**
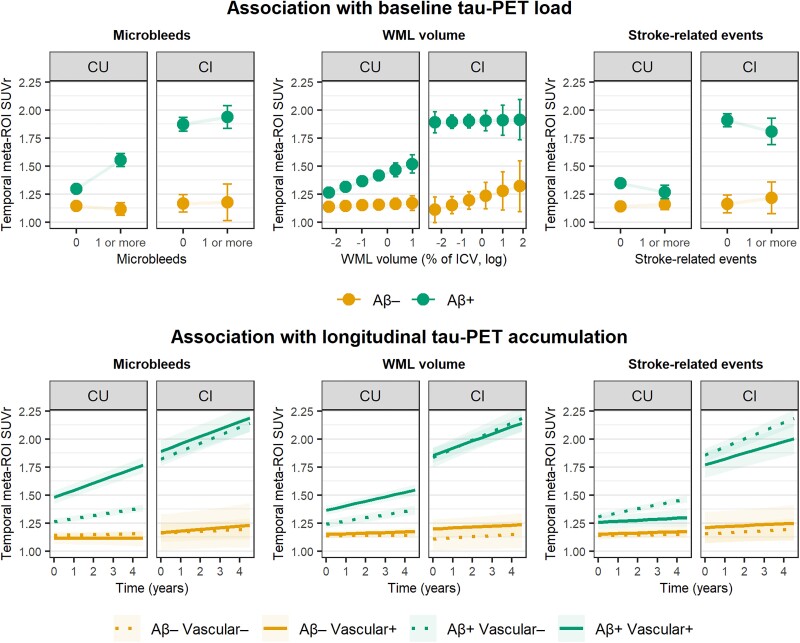
**Interactions between cerebrovascular pathology and amyloid-β pathology on baseline tau load and longitudinal tau accumulation in the Swedish BioFINDER-2 study**. Visualized are the interaction effects between each cerebrovascular factor and amyloid-β (Aβ) on baseline tau load and longitudinal tau accumulation derived from linear mixed models. Estimated baseline and longitudinal tau pathology is shown based on different levels of Aβ and vascular burden for visualization purposes. For cognitively unimpaired (CU) participants, A− reflects the effect for the average amyloid-PET standardized uptake value ratio (SUVr) of Aβ-negative individuals, and A+ reflects the effect for the average amyloid-PET SUVr of Aβ-positive individuals. For cognitively impaired (CI) participants, Aβ− reflects the effect for the average Aβ-negative individual, and Aβ+ reflects the effect for the average Aβ-positive individual. For the longitudinal results with white matter lesion (WML) volume, Vascular− and Vascular+ reflect the effect of −1SD and +1SD from the mean WML volume. For longitudinal results with microbleeds and stroke characteristics, Vascular− and Vascular+ reflect the effect for the average individual with and without microbleeds or stroke characteristics, respectively. ICV = intracranial volume; ROI = region of interest.

Next, we investigated the interaction effect between baseline cerebrovascular pathology and baseline Aβ pathology on both baseline tau load and longitudinal tau accumulation. Estimates and *P*-values are reported in Table 2. For microbleeds, we observed a significant interaction with Aβ load on both baseline tau load (β = 0.68, *P* < 0.001) and longitudinal tau accumulation (β = 0.11, *P* < 0.001) in CU participants (Fig. 2). Thus, CU participants with both high Aβ load and microbleeds showed the greatest baseline tau load and the steepest increases in longitudinal tau accumulation. No significant effects were observed in CI participants. We then tested whether location of microbleeds (i.e. lobar versus non-lobar) influenced the observed association. Overall, observed associations became stronger for microbleeds located in lobar regions, and were non-significant for microbleeds located in non-lobar regions (Table 2). For WML volume, we observed a significant interaction with Aβ load on baseline tau burden in CU participants (β = 0.14, *P* = 0.002) (Fig. 2). We did not observe a significant effect on longitudinal tau, nor did we observe significant effects in CI participants (Table 2). For stroke-related events, we observed a significant interaction effect with Aβ load on longitudinal tau accumulation in CU participants in the opposite direction, such that longitudinal increases in tau accumulation were attenuated in CU participants with both high Aβ load and stroke-related events compared to CU participants with high Aβ load and without stroke-related events (β = −0.08, *P* < 0.001) (Table 2). No significant interaction effects were observed in CI participants. We then examined whether specific sub-components of the stroke characteristics drove this opposite effect, and found significant negative interaction effects between presence of lacunes and Aβ pathology on attenuated longitudinal tau accumulation (Supplementary Fig. 4).

**Table 2:** Interaction effects between cerebrovascular pathology and amyloid-β pathology on baseline tau load and longitudinal tau accumulation

Effect on baseline tau load			
Vascular factor × Aβ	Total sample^a^	Cognitively unimpaired^b^	Cognitively impaired^c^
Microbleeds	β = 0.27, *P* = 0.10	**β = 0.68, *P* < 0.001**	β = 0.10, *P* = 0.54
Lobar microbleeds	**β = 0.40, *P* = 0.01**	**β = 0.79, *P* < 0.001**	β = 0.22, *P* = 0.27
Non-lobar microbleeds	β = −0.30, *P* = 0.22	β = −0.36, *P* = 0.25	β = −0.37, *P* = 0.20
WML volume	β = 0.08, *P* = 0.15	**β = 0.14, *P* = 0.002**	β = −0.06, *P* = 0.50
Stroke-related events	β = −0.09, *P* = 0.52	β = −0.18, *P* = 0.09	β = −0.22, *P* = 0.35

Effect on longitudinal tau accumulation			
Vascular factor × Aβ × Time	Total sample^a^	Cognitively unimpaired^b^	Cognitively impaired^c^
Microbleeds	β = 0.03, *P* = 0.30	**β = 0.11, *P* < 0.001**	β = −0.02, *P* = 0.52
Lobar microbleeds	β = 0.04, *P* = 0.12	**β = 0.11, *P* < 0.001**	β = −0.01, *P* = 0.88
Non-lobar microbleeds	β = −0.03, *P* = 0.47	β = −0.08, *P* = 0.36	β = −0.06, *P* = 0.22
WML volume	β = 0.01, *P* = 0.30	β = 0.01, *P* = 0.12	β = −0.01, *P* = 0.52
Stroke-related events	β = −0.03, *P* = 0.30	**β = −0.08, *P* < 0.001**	β = −0.03, *P* = 0.52

Reported are estimates and *P*-values (false discovery rate-corrected) from linear mixed models with random intercepts. The fixed effect of Vascular-factor × Aβ was interpreted as the effect on baseline tau load, whereas the fixed effect of Vascular-factor × Aβ × Time was interpreted as the effect on longitudinal tau accumulation. Significant associations are highlighted in bold. White matter lesion (WML) volume was corrected for intracranial volume and log-transformed. Continuous variables were scaled within each model. Aβ = amyloid-β.

^a^Model for total sample: tau-PET ∼ Vascular-factor × Aβ-status × Time + Vascular-factor × Aβ-status + Vascular-factor × Time + Aβ-status × Time + Age × Time + Sex × Time + *APOE*-ɛ4 × Time + Cognitive-stage × Time + Vascular-factor + Aβ-status + Time + Age + Sex + *APOE*-ɛ4 + Cognitive-stage.

^b^Model for cognitively unimpaired: tau-PET ∼ Vascular-factor × Aβ-PET-SUVr × Time + Vascular-factor × Aβ-PET-SUVr + Vascular-Factor × Time + Aβ-PET-SUVr × Time + Age × Time + Sex × Time + *APOE*-ɛ4 × Time + Vascular-factor + Aβ-PET-SUVr + Time + Age + Sex + *APOE*-ɛ4.

^c^Model for cognitively impaired: tau-PET ∼ Vascular-factor × Aβ-status × Time + Vascular-factor × Aβ-status + Vascular-factor × Time + Aβ-status × Time + Age × Time + Sex × Time + *APOE*-ɛ4 × Time + Vascular-factor + Aβ-status + Time + Age + Sex + *APOE*-ɛ4.

Since we observed significant interaction effects with Aβ for both WML volume and microbleeds on increased baseline tau load in CU participants, and since participants with microbleeds showed larger WML volumes (*P* < 0.001; Supplementary Fig. 5), we next investigated their independent effects on tau. When including both WML volume and microbleeds as predictors in the same model, the interaction between microbleeds and Aβ load remained strongly associated with both baseline tau load (β = 0.61, *P* < 0.001) and longitudinal tau accumulation (β = 0.12, *P* < 0.001). In contrast, the interaction effect between WML volume and Aβ load on baseline tau load was no longer significant (β = 0.09, *P* = 0.06), and the effect on longitudinal tau remained non-significant (β = −0.01, *P* = 0.31). Finally, to examine whether the significant positive interaction effect between microbleeds and Aβ load may be the result of CU participants with microbleeds having more Aβ pathology and therefore resulting in greater tau burden, we compared the Aβ-PET SUVr values between Aβ-positive CU participants with and without microbleeds. No difference in Aβ-PET SUVr between Aβ-positive CU participants with and without microbleeds were observed (β = 0.01, *P* = 0.71) (Supplementary Fig. 6).

To better interpret the interaction effects, we also performed stratified analyses examining each group independently (Aβ-negative CU, Aβ-positive CU, Aβ-negative CI and Aβ-positive CI; thereby removing the interaction term with Aβ from the models). These stratified analyses revealed highly similar results compared to the original models with an interaction term: in CU Aβ-positive participants, WML volume was associated with increased baseline tau load, microbleeds were associated with both increased baseline tau load and longitudinal tau accumulation, and stroke-related events were associated with decreased longitudinal tau accumulation (all adjusted for age, sex, *APOE* ɛ4 carriership and Aβ-PET SUVr). No significant effects were observed in CU Aβ-negative participants, and also no significant results were observed in CI Aβ-negative and CI Aβ-positive participants (Supplementary Table 3). We also performed two sets of sensitivity analyses. First, in the CI group that had Aβ-PET available (i.e. *n* = 333 MCI and *n* = 16 dementia), we repeated our primary linear mixed models and included continuous Aβ-PET SUVr instead of binary Aβ-status. This revealed similar results (Supplementary Table 4), with the only difference being that stroke-related events now also showed a significant negative interaction effect with Aβ load on baseline tau load in CI individuals (β = −0.25, *P* = 0.03). Second, sensitivity analyses using tau-PET SUVr in a neocortical late-stage tau-region instead of the temporal meta-ROI also revealed comparable results (Supplementary Table 5).

### Role of vascular risk in the association between amyloid-β and tau

We then investigated the interaction effect between vascular risk and Aβ pathology on both baseline tau load and longitudinal tau accumulation. In the total cohort, the FHS-CVD vascular risk score ranged from 2.7% to 97.3% (mean 32.5%) with higher scores representing greater 10-year risk for cardiovascular events. A higher FHS-CVD risk score was associated with older age and male sex (both *P* < 0.001) (Supplementary Fig. 2). Moreover, a higher FHS-CVD risk score was associated with increased WML volume (β = 0.38, *P* < 0.001), more microbleeds (β = 0.03, *P* < 0.001) and more stroke-related events (β = 0.03, *P* < 0.001) (Supplementary Fig. 3).

For the FHS-CVD risk score, we observed significant negative interaction effects with Aβ pathology on baseline tau load in the total sample (β = −0.18, *P* = 0.007) and in CI participants (β = −0.27, *P* = 0.003), and on longitudinal tau accumulation in CU participants (β = −0.02, *P* = 0.02) (Supplementary Table 6). Sensitivity analyses excluding age and sex as covariates from the model yielded identical results (Supplementary Table 6). Thus, participants with high Aβ pathology and high vascular risk showed attenuated tau accumulation. Secondary analyses were performed to test whether this was explained by specific subcomponents of the FHS-CVD risk score. We observed that the negative interaction effect was explained by subcomponents younger age, female sex and lower BMI, which all showed significant negative interactions with Aβ pathology on baseline tau load and/or longitudinal tau accumulation (Supplementary Table 6). Comparable results were observed using tau-PET SUVr in a neocortical late-stage tau-region (Supplementary Table 5).

### Neuropathological replication cohort

**Figure 3:**
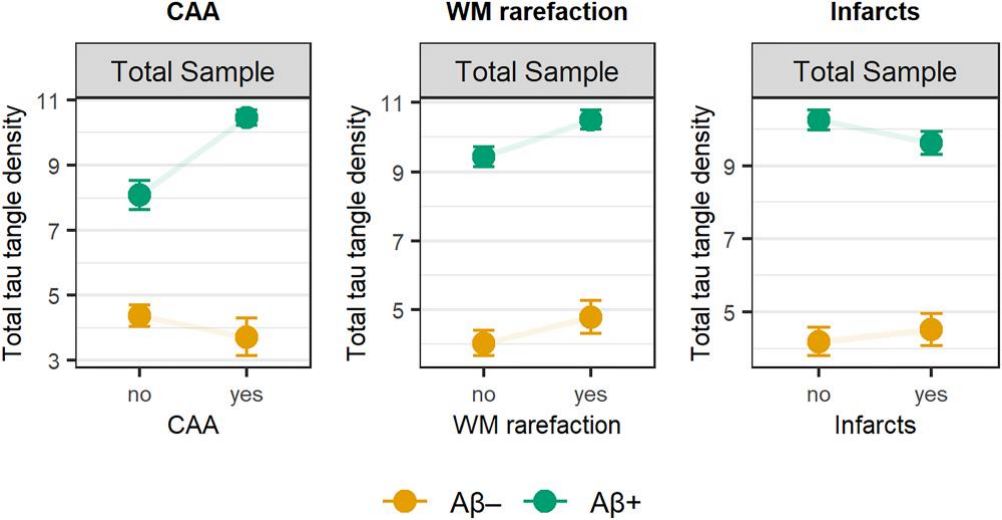
**Interactions between cerebrovascular pathology and amyloid-β plaque density on tau tangle density in the Arizona-based neuropathology cohort**. Visualized are the interaction effects between each cerebrovascular factor and amyloid-β plaque density on tau tangle density derived from linear models. Estimated tau tangle density is shown based on different levels of plaque density: A− reflects the effect for the average plaque density of amyloid-β low individuals (i.e. CERAD non-to-sparse), and A+ reflects the effect for the average plaque density of amyloid-β high individuals (i.e. CERAD moderate-to-frequent). Aβ = amyloid-β; CAA = cerebral amyloid angiopathy; CERAD = Consortium to Establish a Registry for Alzheimer’s Disease; WM = white matter.

Finally, we aimed to validate the results observed *in vivo* for cerebrovascular pathology (WML volume, microbleeds and stroke-characteristics) using data from the Arizona-based neuropathology cohort (WM rarefaction, CAA and infarcts). A total of 1610 participants were included, among which 510 (31.7%) low-Aβ participants and 1100 (68.3%) high-Aβ participants. Participant characteristics are shown in Table 3. For CAA, a significant positive interaction effect with Aβ plaque density on tau tangle density was observed (β = 0.38, *P* < 0.001) (Fig. 3). In a subset of 1109 participants with a semi-quantitative (instead of dichotomous) measure of CAA available, we also observed a significant CAA-by-Aβ plaque density interaction on tau tangle density (β = 0.55, *P* < 0.001). Thus, participants with both high Aβ plaque density and CAA showed the greatest tau tangle density. For WM rarefaction, we did not observe a significant interaction effect with Aβ plaque density on tau tangle density (β = 0.04, *P* = 0.37). For infarcts, we observed a significant negative interaction effect with Aβ plaque density on tau tangle density (β = −0.11, *P* = 0.005), thus participants with both high Aβ plaque density and infarcts showed attenuated tau tangle density. Overall, these findings using neuropathological data from an independent cohort largely confirmed our *in vivo* observed results.

**Table 3:** Demographics of Arizona-based neuropathology cohort

	Total sample
*n*	1610
Expired age, years	82.2 ± 8.6
Sex, *n* female (%)	696 (43.2)
*APOE* ɛ4 status, *n* carrier (%)	601 (37.3)
MMSE last known	18.8 ± 9.4
**Cerebrovascular pathology**	
CAA, *n* yes (%)	953 (59.2)
WM rarefaction, *n* yes (%)	745 (46.3)
Infarcts, *n* yes (%)	700 (43.5)
**Amyloid-β and tau**	
CERAD, *n* moderate-to-frequent (%)	1100 (68.3)
Amyloid-β plaque density total	8.8 ± 5.9
Tau tangle density total	8.1 ± 4.7

Values shown are mean ± standard deviation, unless specified otherwise. CAA = cerebral amyloid angiopathy; CERAD = Consortium to Establish a Registry for Alzheimer’s Disease; MMSE = Mini-Mental State Examination; WM = white matter.

## Discussion

This study aimed to investigate whether vascular burden interacts with Aβ on both baseline tau tangle load and longitudinal tau accumulation in 1229 participants from the BioFINDER-2 study. We observed differential effects on tau accumulation depending on the vascular factor. In cognitively unimpaired (but not in impaired) individuals, the co-presence of microbleeds and Aβ pathology was associated with greater baseline tau load and steeper longitudinal increases in tau. An opposite effect was observed for stroke-related events (which was driven by lacunes), such that unimpaired individuals with increased Aβ pathology and stroke-related events showed attenuated longitudinal tau accumulation compared to individuals with increased Aβ pathology and no stroke-related events. We did not observe a robust interaction effect between WML volume and Aβ pathology on tau accumulation. Neuropathological data from 1610 autopsy cases validated the presence of a positive interaction effect between CAA and Aβ, and the presence of a negative interaction effect between infarcts and Aβ, on tau tangle density. Finally, we observed significant negative interaction effects between the FHS-CVD vascular risk score and Aβ on attenuated tau accumulation, which was explained by sub-components younger age, female sex and lower BMI. Together, our results suggest that cerebrovascular pathology (in the presence of Aβ pathology) can modify tau accumulation in early AD stages, which is of interest for clinical trial designs.

A key finding was that the co-presence of microbleeds and Aβ pathology was associated with accelerated tau accumulation in early stages of the disease. We validated this observation using neuropathological data from an independent cohort, showing that the co-presence of CAA and Aβ plaque density was associated with increased tau tangle density. This is in line with a recent study which also showed a synergistic influence of CAA and Aβ plaque burden on tau tangle burden at autopsy in a different neuropathology cohort.^18^ Moreover, an association between a quantitative measure of CAA and tau Braak stage independent from Aβ load has previously been reported in a subset of the Arizona-based neuropathology cohort that was used in the current study.^22^ CAA (i.e. the build-up of Aβ within the walls of blood vessels in the brain) is primarily characterized on MRI by the presence of haemorrhagic lesions in lobar regions.^43^ This corresponds to our finding that the interaction effect between Aβ and microbleeds on tau pathology became stronger when examining microbleeds located in lobar regions, further strengthening the comparability of our *in vivo* and post-mortem findings. Importantly, the presence of microbleeds was associated with increased tau accumulation only in Aβ-positive (and not Aβ-negative) individuals, indicating that microbleeds only influence tau in the presence of Aβ pathology. Furthermore, our results showed that although Aβ-positive individuals had more microbleeds than Aβ-negative individuals, individuals with microbleeds did not have more Aβ-pathology than individuals without microbleeds. Thus, it is unlikely that the significant interaction effect between Aβ and microbleeds on tau pathology is driven solely by effects of Aβ plaque load. The underlying mechanism through which lobar microbleeds (or CAA) interact with Aβ resulting in increased tau accumulation remains unclear. CAA may lead to reduced clearance of soluble Aβ isoforms, thereby resulting in an increased concentration and altered composition of soluble Aβ isoforms in the brain. This could affect tau accumulation directly or via mechanisms including blood–brain barrier disruption and/or the activation of neuro-inflammatory responses, both of which have been associated with tau.^44,45^

Although we found a significant interaction effect between WML volume and Aβ with baseline tau burden in CU individuals, this association was no longer significant when accounting for effects of microbleeds. Similar to our *in vivo* results, we did not find an interaction between WM rarefaction and Aβ plaque density with tau tangle density in the neuropathology cohort. In a previous study, associations between WML volume and tau accumulation in individuals without dementia were also non-significant.^26^ Moreover, we did not observe significant differences in WML volume between Aβ-negative and Aβ-positive individuals at the same cognitive stage, but we did observe a significant difference in WML volume between unimpaired and impaired participants irrespective of Aβ status. Together, this suggests that WML volume is likely not strongly associated with AD pathology, even though it is associated with cognitive impairment.^46^ The aetiology of WML is not fully understood, and previous studies have indicated that the aetiology may differ depending on the lobar location of the lesions.^47^ Examining the influence of regional WML (in interaction with Aβ) on tau accumulation as well as other imaging measures of white matter damage (e.g. diffusion tensor imaging) would be of interest for future research.

With regards to stroke-related events, we observed a significant negative interaction effect between the presence of stroke-related events and higher Aβ, resulting in attenuated tau accumulation in very early stages of the disease. This negative interaction effect was driven by effects of lacunes. A previous study with CSF biomarkers also found that the presence of lacunes was associated with less Aβ_42_ and tau pathology (in contrast to microbleeds, which was associated with more Aβ and tau pathology).^48^ There are several potential explanations for the negative association between lacunes and tau. First, it could be that stroke-related events and lacunes are more detrimental for cognitive functioning (compared to e.g. microbleeds), and therefore this effect could represent a survival bias, i.e. individuals that have both stroke-related events and Aβ pathology are less likely to remain cognitively unimpaired.^14,49^ An alternative explanation is related to the hypothesis that tau spreads via structurally or functionally connected neurons.^50,51^ It could be that lacunes lead to disrupted brain connectivity, thereby limiting the spread of tau throughout the brain. Both of these potential mechanisms require further investigation.

Interestingly, we observed significant negative interaction effects between higher FHS-CVD risk (reflecting greater 10-year risk for future cardiovascular events) and higher Aβ resulting in attenuated tau accumulation. This is in contrast with previous studies, which observed positive interaction effects.^16,17^ In our data, the negative effect was explained by the subcomponents younger age, female sex and lower BMI. Although younger age and female sex generally reflect a lower vascular risk,^38^ multiple studies have shown that younger age and female sex are associated with increased tau pathology in people with AD.^52-55^ Similarly, although lower BMI generally reflects a lower vascular risk,^38^ a decrease in BMI is frequently observed in AD patients with MCI and dementia.^56^ Therefore, this composite vascular risk score may not always optimally reflect vascular risk in a study population with (clinical) AD as it may be confounded by variables more closely associated to AD-related pathological and clinical processes. It is possible that other vascular risk factors and vascular risk measured at mid-life (prior to the accumulation of tau) provides a better relationship with AD pathology at older age, as has also been suggested by previous studies,^57^ which would be of interest for a future study.

Importantly, our key finding in the *in vivo* analyses of microbleeds accelerating tau accumulation in the presence of Aβ pathology was found in the early disease population. This suggests that microbleeds play a role on the very early interplay between Aβ and tau but are not a substantial contributor to tau accumulation in later disease stages. This is valuable information for prevention strategies and clinical trial designs, as it opens the possibility that (additionally) targeting microbleeds in preclinical stages may attenuate the rate of early tau accumulation and thereby slow down the pathological progression of the disease. Although targeting microbleeds in clinical stages of the disease may have less effect on tau accumulation, further research is needed to conclude on whether targeting cerebrovascular pathology in clinical stages could nevertheless still result in attenuated clinical progression.

Strengths of this study include the use of two large and well-characterized cohorts: one *in vivo* biomarker-focused cohort including measures of vascular burden, Aβ-PET and (longitudinal) tau-PET, and one independent post-mortem cohort used to validate the *in vivo* results. Furthermore, several measures of vascular burden were investigated, including MRI measures of cerebrovascular pathology, vascular risk and neuropathological measures of cerebrovascular pathology. However, this study also had limitations. First, a CSF-based dichotomous measure of Aβ-pathology was used for dementia participants, since (per protocol) the majority of patients with dementia did not undergo Aβ-PET, which may have led to a less accurate investigation of Aβ effects on tau in the total CI group. Second, the microbleeds and stroke-related events were all visually rated by a single highly experienced radiologist, and we acknowledge that a consensus visual read between multiple readers would be more reliable. Third, potential differences between the *in vivo* and neuropathology cohort in terms of number of CU and CI individuals, measurement of cerebrovascular pathology and underlying aetiologies in CI Aβ-negative individuals may limit comparability of the results. Fourth, we did not have cholesterol data available, and we were therefore not able to calculate the lipid-based FHS-CVD risk score.^38^ The lipid-based score does not include BMI as a subcomponent, and may therefore be better suitable for this population, which would be of interest to investigate in further studies.

## Conclusion

Our results suggest that, in the presence of Aβ pathology, cerebrovascular pathology modifies tau accumulation in early stages of the disease. A key finding was that the co-occurrence of microbleeds and amyloid-β pathology was associated with greater accumulation of tau aggregates during early disease stages. This opens the possibility that (additionally) targeting microbleeds may attenuate the rate of initial tau accumulation as a means to slow down the progression of the disease.

## Supplementary Material

awad317_Supplementary_Data

## Data Availability

Anonymized data from BioFINDER will be shared on request from a qualified academic investigator for the sole purpose of replicating procedures and results presented in the article and as long as data transfer is in agreement with EU legislation on the general data protection regulation and decisions by the Swedish Ethical Review Authority and Region Skåne, which should be regulated in a material transfer agreement. Anonymized data from the Arizona Study of Aging and Neurodegenerative Disorders/Brain and Body Donation Program study will be shared by request from a qualified academic investigator and as long as data transfer is in agreement with USA legislation on the general data protection regulation and decisions by the Institutional Review Boards of the Brain and Body Donation Program and the Arizona Study of Aging and Neurodegenerative Disorders.
