# Supplementary material for: Interactions between vascular burden and amyloid-β pathology on trajectories of tau accumulation
Source: Brain. 2023 Sep 18;147(3):949–60. doi: 10.1093/brain/awad317 (PMC10907085; doi:10.1093/brain/awad317)
Supplement: awad317_Supplementary_Data [file awad317_supplementary_data.pdf]

Supplemental Materials

Supplementary Table 1 Clinical diagnoses of Aβ-negative cognitively impaired participants

Diagnosis	N
Mild cognitive impairment	131
Dementia with Lewy Bodies	18
Vascular dementia	15
Behavioural variant frontotemporal dementia	14
Semantic variant primary progressive aphasia	2
Primary progressive aphasia	1
Progressive supranuclear palsy	17
Corticobasal syndrome	4
Multiple system atrophy	6
Parkinson's disease	24
Parkinsonism (not otherwise specified)	5
Neurodegenerative disorder (not otherwise specified)	6
Not determined	14

Supplementary Table 2 Between-group differences in cerebrovascular pathology

	**Microbleeds**	**Lobar microbleeds**	**Non-lobar microbleeds**	**WML volume**	**Stroke-related** events
			**A+ vs. A- participants from the same cognitive stage**		
CU A+ vs. CU A-	**β=0.66, *p*=0.04**	**β=1.03, *p*=0.01**	β=-0.55, *p*=0.50	β=0.03, *p*=0.68	β=-0.07, *p*=0.83
CI A+ vs. CI A-	**β=0.51, *p*=0.04**	**β=0.74, *p*=0.01**	β=-0.10, *p*=0.81	β=0.08, *p*=0.25	β=-0.10, *p*=0.80
			**CI vs. CU participants**		
CI A- vs. CU A-	**β=0.70, *p*=0.03**	**β=0.76, *p*=0.03**	β=0.52, *p*=0.47	**β=0.28, *p*<0.001**	β=0.47, *p*=0.18
CI A+ vs. CU A-	**β=1.21, *p*<0.001**	**β=1.50, *p*<0.001**	β=0.43, *p*=0.47	**β=0.36, *p*<0.001**	β=0.38, *p*=0.18
CI A- vs. CU A+	β=0.04, *p*=0.90	β=-0.27, *p*=0.43	β=1.08, *p*=0.36	**β=0.24, *p*=0.004**	β=0.54, *p*=0.18
CI A+ vs. CU A+	**β=0.55, *p*=0.04**	β=0.47, *p*=0.10	β=0.98, *p*=0.36	**β=0.33, *p*<0.001**	β=0.45, *p*=0.18

Reported are unstandardized estimates from linear regressions (for WML volume) or logistic (for microbleeds and stroke-related events) regressions corrected for age, sex and *APOE* ε4 carriership with estimated marginal means for between-group post-hoc pairwise multiple comparisons. *P*-values are corrected using the False Discovery Rate. WML volume is corrected for ICV and log-transformed.

Supplementary Table 3 Effects of cerebrovascular pathology on baseline tau load and longitudinal tau accumulation stratified for each group

	**Effect on baseline tau load**			
**Vascular factor**	**CU A+[a]**	**CU A-[a]**	**CI A+[b]**	**CI A-[b]**
WML volume	**β=0.25, *p*=0.008**	β=-0.04, *p*=0.52	β=0.04, *p*=0.38	β=0.01, *p*=0.98
Microbleeds	**β=0.57, *p*=0.007**	β=0.01, *p*=0.97	β=0.14, *p*=0.20	β=-0.25, *p*=0.14
Stroke-related events	β=-0.04, *p*=0.86	β=-0.16 *p*=0.34	β=-0.09, *p*=0.49	β=-0.01, *p*=0.93
	**Effect on longitudinal tau accumulation**			
**Vascular factor*time**	**CU A+[a]**	**CU A-[a]**	**CI A+[b]**	**CI A-[b]**
WML volume	β=0.03, *p*=0.08	β=-0.01, *p*=0.75	β=-0.01, *p*=0.37	β=-0.03, *p*=0.23
Microbleeds	**β=0.11, *p*=0.003**	β=0.02, *p*=0.68	β=-0.01, *p*=0.68	β=0.03, *p*=0.56
Stroke-related events	**β=-0.08, *p*=0.04**	β=0.03, *p*=0.39	β=-0.03, *p*=0.10	β=-0.02, *p*=0.60

Reported are estimates and p-values from linear mixed models with random intercepts. The fixed effect of vascular-factor was interpreted as the effect on baseline tau load, whereas the fixed effect of vascular-factor*time was interpreted as the effect on longitudinal tau accumulation. WML volume was corrected for ICV and log-transformed. Continuous variables were scaled within each model.

[a]Model for CU: tau-PET SUVr ~ vascular-factor*time + Aβ-PET-SUVR*time + age*time + sex*time + APOE-ε4*time + vascular-factor + Aβ-PET-SUVR + age + sex + APOE-ε4

[b]Model for CI: tau-PET SUVr ~ vascular-factor*time + age*time + sex*time + APOE-ε4*time + vascular-factor + age + sex + APOE-ε4

Supplementary Table 4 Interaction effects of cerebrovascular pathology and amyloid-β PET SUVr (instead of amyloid-β status) on baseline tau load and longitudinal tau accumulation in the subset of CI participants that had amyloid-β PET available

	**Effect on baseline tau load**
**Vascular factor*Aβ**	**CI**
WML volume	β=-0.06, *p*=0.19
Microbleeds	β=-0.07, *p*=0.51
Lobar microbleeds	β=-0.09, *p*=0.51
Non-lobar microbleeds	β=-0.73, *p*=0.69
Stroke-related events	**β=-0.25, *p*=0.03**
	**Effect on longitudinal tau accumulation**
**Vascular factor*Aβ*time**	**CI**
WML volume	β=-0.01, *p*=0.20
Microbleeds	β=-0.02, *p*=0.53
Lobar microbleeds	β=0.01, *p*=0.92
Non-lobar microbleeds	β=-0.05, *p*=0.15
Stroke-related events	β=-0.03, *p*=0.22

Reported are estimates and p-values from linear mixed models with random intercepts. The fixed effect of vascular-factor*Aβ was interpreted as the effect on baseline tau load, whereas the fixed effect of vascular-factor*Aβ*time was interpreted as the effect on longitudinal tau accumulation. WML volume was corrected for ICV and log-transformed. Continuous variables were scaled within each model.

Model: tau-PET SUVr ~ vascular-factor*Aβ-PET-SUVr*time + vascular-factor*Aβ-PET-SUVr + vascular-factor*time + Aβ-PET-SUVr*time + age*time + sex*time + APOE-ε4*time + vascular-factor + Aβ-PET-SUVr + time + age + sex + APOE-ε4

Supplementary Table 5 Interaction effects between cerebrovascular pathology, vascular risk and amyloid-β pathology on baseline tau load and longitudinal tau accumulation in a late-stage tau-region (Braak V-VI)

	**Effect on baseline tau load**		
**Vascular factor*Aβ**	**Total Sample[a]**	**CU[b]**	**CI[c]**
Microbleeds	β=0.08, *p*=0.59	**β=0.37, *p*=0.01**	β=-0.01, *p*=0.97
WML volume	β=-0.03, *p*=0.59	β=0.04, *p*=0.32	**β=-0.14, *p*=0.04**
Stroke-related events	**β=-0.32, *p*=0.04**	β=-0.15, *p*=0.24	**β=-0.43, *p*=0.02**
FHS-CVD risk score	**β=-0.28, *p*<0.001**	β=-0.07, *p*=0.24	**β=-0.37, *p*<0.001**
	**Effect on longitudinal tau accumulation**		
**Vascular factor*Aβ*time**	**Total Sample[a]**	**CU[b]**	**CI[c]**
Microbleeds	β=0.02, *p*=0.53	**β=0.12, *p*=0.004**	β=-0.04, *p*=0.34
WML volume	β=-0.01, *p*=0.85	β=0.01, *p*=0.20	β=-0.02, *p*=0.10
Stroke-related events	β=-0.03, *p*=0.26	**β=-0.09, *p*=0.01**	β=-0.05, *p*=0.19
FHS-CVD risk score	**β=-0.03, *p*=0.03**	**β=-0.03, *p*=0.01**	β=-0.04, *p*=0.08

Reported are estimates and p-values (FDR-corrected) from linear mixed models with random intercepts. The fixed effect of vascular-factor*Aβ was interpreted as the effect on baseline tau load, whereas the fixed effect of vascular-factor*Aβ*time was interpreted as the effect on longitudinal tau load. WML volume was corrected for intracranial volume and log-transformed. Continuous variables were scaled within each model. Aβ = amyloid-β; CI = cognitively impaired; CU = cognitively unimpaired; WML = white matter lesion.

[a] Model for Total Sample: tau-PET ~ vascular-factor*Aβ-status*time + vascular-factor*Aβ-status + vascular-factor*time + Aβ-status*time + age*time + sex*time + APOE-ε4*time + cognitive-stage*time + vascular-factor + Aβ-status + time + age + sex + APOE-ε4 + cognitive-stage

[b] Model for CU: tau-PET ~ vascular-factor*Aβ-PET-SUVr*time + vascular-factor*Aβ-PET-SUVr + vascular-factor*time + Aβ-PET-SUVr*time + age*time + sex*time + APOE-ε4*time + vascular-factor + Aβ-PET-SUVr + time + age + sex + APOE-ε4

[c] Model for CI: tau-PET ~ vascular-factor*Aβ-status*time + vascular-factor*Aβ-status + vascular-factor*time + Aβ-status*time + age*time + sex*time + APOE-ε4*time + vascular-factor + Aβ-status + time + age + sex + APOE-ε4

Supplementary Table 6 Interaction effects of vascular risk and amyloid-β pathology on baseline tau load and longitudinal tau accumulation

	Effect on baseline tau load		
**Vascular factor*Aβ**	**Total Sample**	**CU**	**CI**
FHS-CVD risk score	**β=-0.18, *p*=0.007**	β=-0.04, *p*=0.36	**β=-0.27, *p*=0.003**
FHS-CVD risk score (without correcting for age and sex)	**β=-0.19, *p*=0.001**	β=-0.06, *p*=0.27	**β=-0.27, *p*<0.001**
Age	**β=-0.14, *p*=0.008**	**β=-0.11, *p*=0.02**	**β=-0.25, *p*<0.001**
Sex (female)	β=0.17, *p*=0.07	**β=0.16, *p*=0.03**	**β=0.27, *p*=0.048**
BMI	**β=-0.20, *p*<0.001**	**β=-0.22, *p*<0.001**	β=-0.14, *p*=0.054
SBP	β=-0.05, *p*=0.42	β=0.02, *p*=0.76	β=-0.05, *p*=0.49
Smoking (yes)	β=-0.01, *p*=0.99	β=-0.14, *p*=0.39	β=0.04, *p*=0.88
Diabetes (yes)	β=-0.07, *p*=0.69	**β=0.63, *p*<0.001**	β=-0.29, *p*=0.17
	**Effect on longitudinal tau accumulation**		
**Vascular factor*Aβ*time**	**Total Sample**	**CU**	**CI**
FHS-CVD risk score	β=-0.01, *p*=0.30	**β=-0.02, *p*=0.02**	β=-0.01, *p*=0.52
FHS-CVD risk score (without correcting for age and sex)	β=-0.01, *p*=0.21	**β=-0.02, *p*=0.01**	β=-0.01, *p*=0.34
Age	β=-0.01, *p*=0.86	**β=-0.02, *p*=0.005**	β=-0.01, *p*=0.33
Sex (female)	β=0.01, *p*=0.97	β=0.01, *p*=0.66	β=0.01, *p*=0.84
BMI	**β=-0.04, *p*<0.001**	**β=-0.02, *p*=0.04**	**β=-0.05, *p*<0.001**
SBP	**β=0.02, *p*=0.01**	**β=0.03, *p*<0.001**	β=0.02, *p*=0.19
Smoking (yes)	β=-0.02, *p*=0.49	**β=-0.07, *p*=0.04**	β=0.06, *p*=0.31
Diabetes (yes)	β=-0.02, *p*=0.23	β=-0.01, *p*=0.89	β=-0.04, *p*=0.25

Reported are estimates and p-values from linear mixed models with random intercepts. The fixed effect of vascular-factor*Aβ was interpreted as the effect on baseline tau load, whereas the fixed effect of vascular-factor*Aβ*time was interpreted as the effect on longitudinal tau accumulation. Continuous variables were scaled within each model. An additional model was performed for the FHS-CVD risk score without correcting for age and sex (and their interaction with time) since age and sex are also subcomponents of the FHS-CVD risk score itself.

Supplementary Figure 1 Tau-PET temporal meta-ROI SUVr in each group

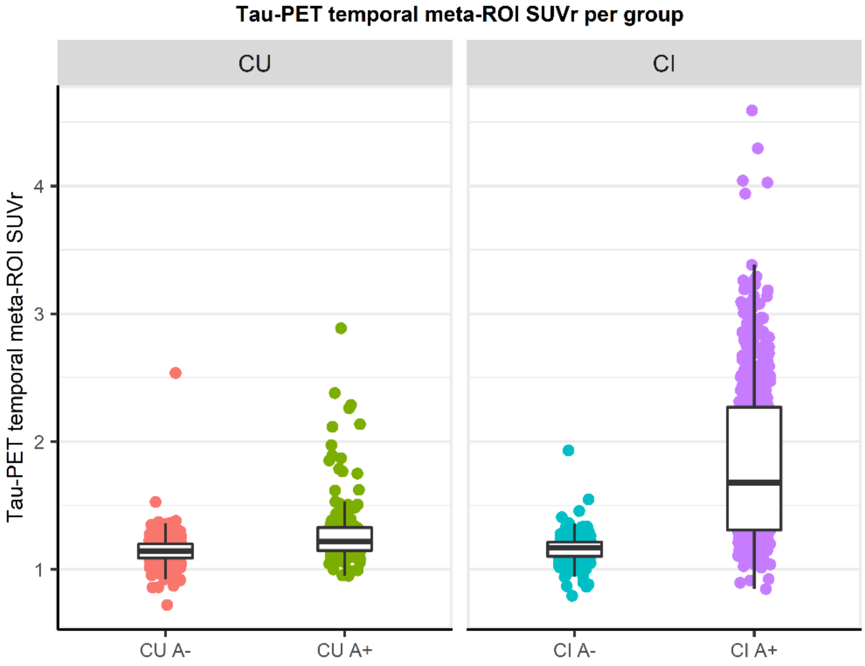

There was 1 amyloid-PET negative CU participant (CU A-) with very high tau load (temporal meta-ROI: 2.54 SUVr), which acted as a statistical outlier in analyses restricted to the amyloid-β negative CU group. Since this participant was a statistical outlier, we excluded this participant from further analyses.

Supplementary Figure 2 Associations between cerebrovascular pathology and vascular risk with age and sex

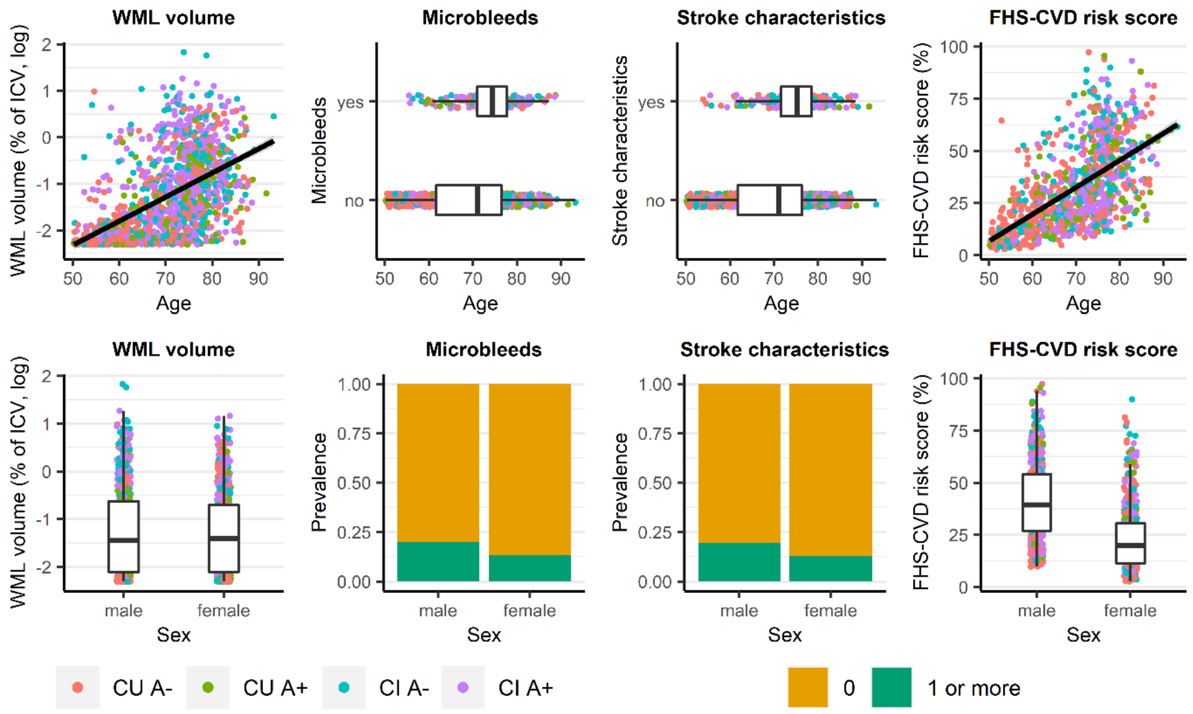

We performed linear (for WML volume and the FHS-CVD risk score) and logistic (for microbleeds and stroke-related events) regressions between age and sex (predictors, separate models) and each vascular factor (outcome, separate models). Older age was associated with increased WML volume (β=0.52, $p<0.001$), presence of microbleeds (β=0.06, $p<0.001$), presence of stroke-related events (β=0.08, $p<0.001$) and a higher FHS-CVD risk score (β=0.60, $p<0.001$). Male sex was significantly associated with presence of microbleeds (β=0.49, $p=0.002$), presence of stroke-related events (β=0.51, $p=0.001$) and a higher FHS-CVD risk score (β=0.46, $p<0.001$), but not with WML volume (β=0.02, $p=0.48$).

Supplementary Figure 3 Associations between vascular risk and cerebrovascular pathology

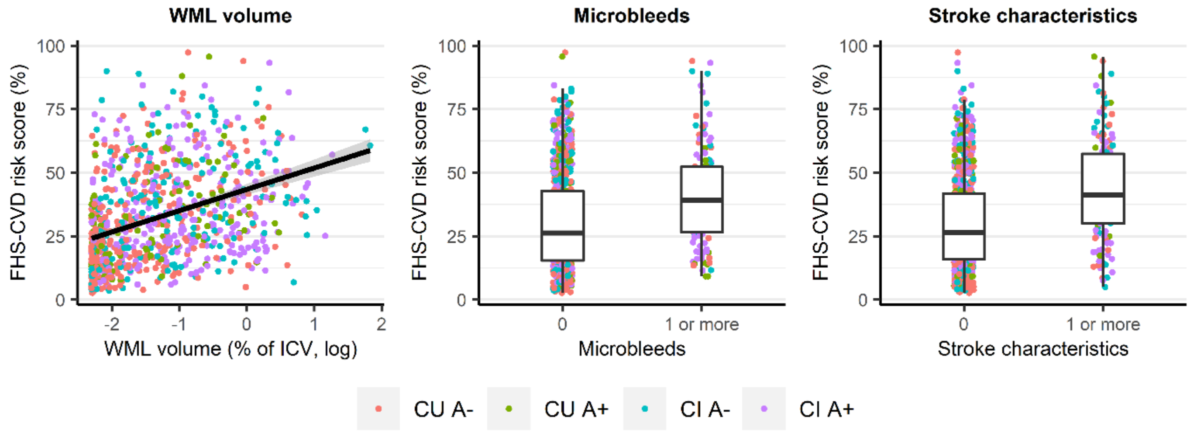

We performed linear regressions between WML volume, microbleeds and stroke-related events (predictors, separate models) and the FHS-CVD risk score (outcome). A higher FHS-CVD risk score was associated with increased WML volume (β=0.38, p<0.001), more microbleeds (β=0.03, p<0.001), and more stroke-related events (β=0.03, p<0.001).

Supplementary Figure 4 Interactions between lacunes and amyloid-β pathology on baseline tau load and longitudinal tau accumulation

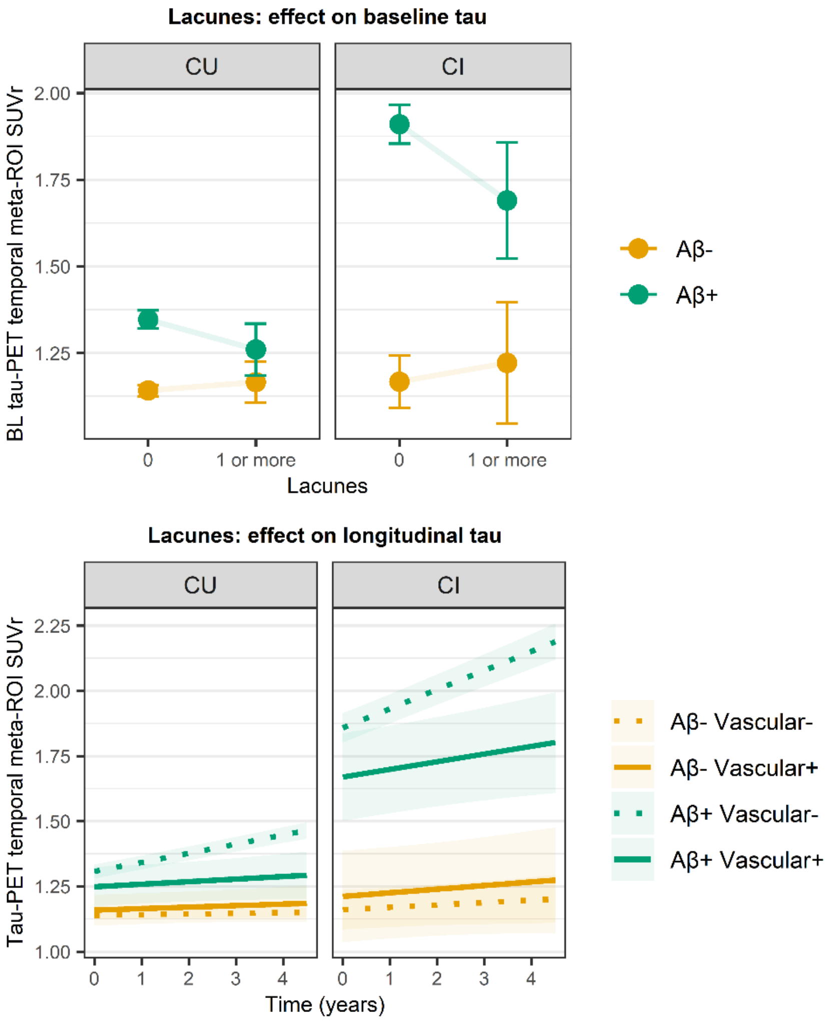

There was a significant negative interaction effect between lacunes and amyloid-β pathology on longitudinal tau accumulation in CU (β=-0.08, *p*=0.001) and CI participants (β=-0.08, *p*=0.01). Interaction effects between lacunes and amyloid-β pathology on baseline tau load were trend-level significant in CU (β=-0.22, *p*=0.06) and CI participants (β=-0.38, *p*=0.07).

Supplementary Figure 5 Differences between participants without microbleeds and participants with microbleeds in WML volume

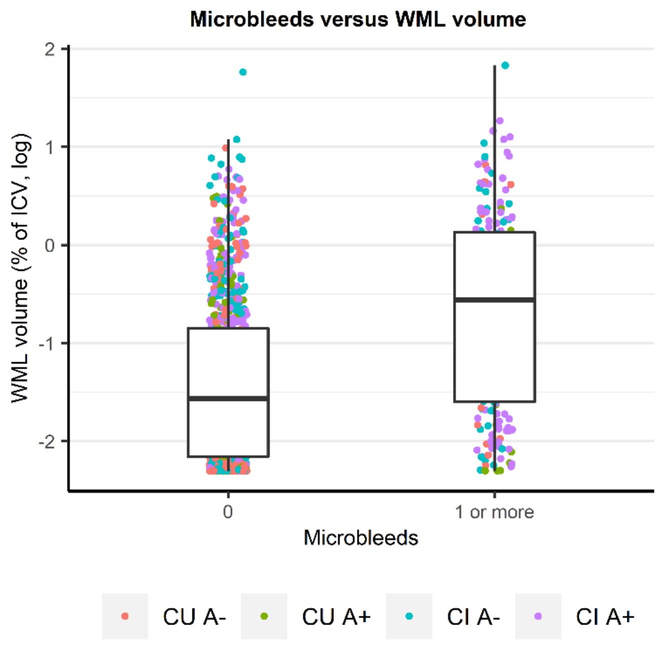

Participants with microbleeds showed significantly larger WML volumes ($p<0.001$)

Supplementary Figure 6 Differences between CU participants with and without amyloid pathology and with and without microbleeds in amyloid-PET SUVr

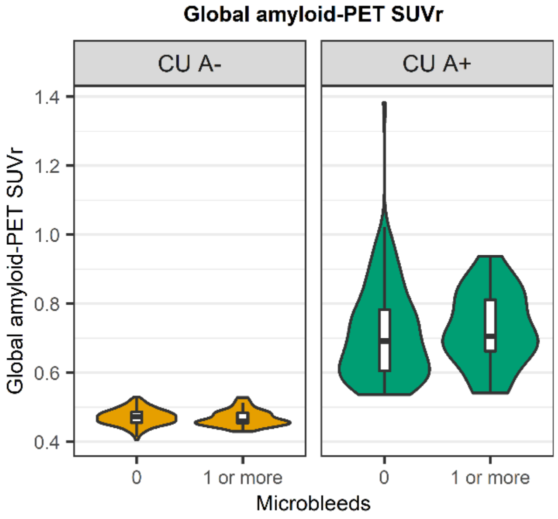

CU participants with microbleeds did not show higher amyloid-PET SUVr compared to CU participants without microbleeds (*p*=0.71).

Supplementary Figure 7 Interactions between the FHS-CVD vascular risk score and amyloid-β pathology on baseline tau load and longitudinal tau accumulation

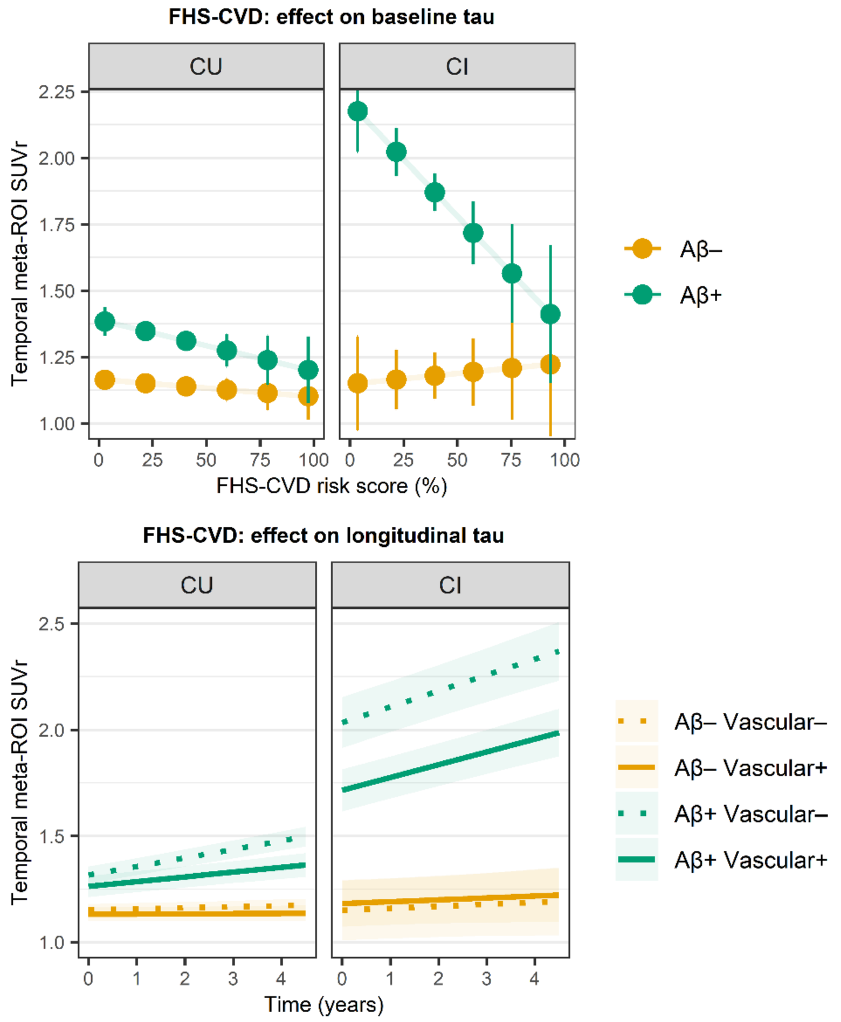

There was a significant negative interaction effect between the FHS-CVD risk score and amyloid-β pathology on baseline tau load in CI participants (β=-0.27, *p*=0.003) and a significant negative interaction effect between the FHS-CVD risk score and amyloid-β pathology on longitudinal tau accumulation in CU participants (β=-0.02, *p*=0.02).
